# (4-Chloro­phen­yl)[1-(4-methoxy­phen­yl)-3-(5-nitro-2-fur­yl)-1*H*-pyrazol-4-yl]methanone

**DOI:** 10.1107/S1600536810011931

**Published:** 2010-04-14

**Authors:** Jia Hao Goh, Hoong-Kun Fun, Nithinchandra, B. Kalluraya, N. Satheesh Rai

**Affiliations:** a X-ray Crystallography Unit, School of Physics, Universiti Sains Malaysia, 11800 USM, Penang, Malaysia; b Department of Studies in Chemistry, Mangalore University, Mangalagangotri, Mangalore 574 199, India

## Abstract

In the title compound, C_21_H_14_ClN_3_O_5_, an intra­molecular C—H⋯O hydrogen bond generates an *S*(7) ring motif and the furan and pyrazole rings are almost coplanar, making a dihedral angle of 1.98 (5)°. The pyrazole ring is inclined at dihedral angles of 47.59 (4) and 7.27 (4)° to the chloro­phenyl and methoxy­phenyl groups, respectively. The nitro group is almost coplanar to its attached furan ring [dihedral angle = 2.03 (12)°]. In the crystal, inter­molecular C—H⋯O hydrogen bonds link the mol­ecules into a three-dimensional network. The crystal structure also features short inter­molecular O⋯N [2.8546 (12) Å] and Cl⋯O [3.0844 (9) Å] contacts as well as aromatic π–π stacking inter­actions [centroid–centroid distance = 3.4367 (6) Å].

## Related literature

For general background to and applications of the title compound, see: Hedge *et al.* (2006 ▶); Kalluraya *et al.* (1994 ▶); Rai & Kalluraya (2006 ▶); Rai *et al.* (2008 ▶). For graph-set theory, see: Bernstein *et al.* (1995 ▶). For closely related structures, see: Goh *et al.* (2009**a* ▶,b* ▶, 2010 ▶). For the stability of the temperature controller used for the data collection, see: Cosier & Glazer (1986 ▶).
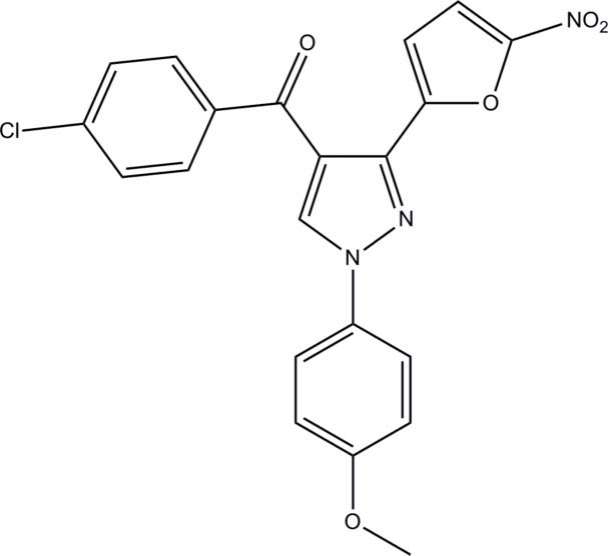


## Experimental

### 

#### Crystal data

C_21_H_14_ClN_3_O_5_
*M* *_r_* = 423.80
Triclinic, 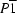

*a* = 9.5589 (8) Å
*b* = 9.6603 (8) Å
*c* = 10.6401 (9) Å
α = 95.523 (2)°
β = 91.074 (2)°
γ = 107.706 (2)°
*V* = 930.44 (13) Å^3^
*Z* = 2
Mo *K*α radiation
μ = 0.25 mm^−1^
*T* = 100 K
0.35 × 0.30 × 0.15 mm

#### Data collection

Bruker SMART APEX DUO CCD diffractometer
Absorption correction: multi-scan (*SADABS*; Bruker, 2009 ▶) *T*_min_ = 0.919, *T*_max_ = 0.963
31674 measured reflections
8076 independent reflections
7107 reflections with *I* > 2σ(*I*)
*R*_int_ = 0.026

#### Refinement

*R*[*F* ^2^ > 2σ(*F* ^2^)] = 0.036
*wR*(*F* ^2^) = 0.146
*S* = 1.13
8076 reflections
272 parameters
H-atom parameters constrained
Δρ_max_ = 0.87 e Å^−3^
Δρ_min_ = −0.70 e Å^−3^

### 

Data collection: *APEX2* (Bruker, 2009 ▶); cell refinement: *SAINT* (Bruker, 2009 ▶); data reduction: *SAINT*; program(s) used to solve structure: *SHELXTL* (Sheldrick, 2008 ▶); program(s) used to refine structure: *SHELXTL*; molecular graphics: *SHELXTL*; software used to prepare material for publication: *SHELXTL* and *PLATON* (Spek, 2009 ▶).

## Supplementary Material

Crystal structure: contains datablocks global, I. DOI: 10.1107/S1600536810011931/hb5382sup1.cif

Structure factors: contains datablocks I. DOI: 10.1107/S1600536810011931/hb5382Isup2.hkl

Additional supplementary materials:  crystallographic information; 3D view; checkCIF report

## Figures and Tables

**Table 1:** Hydrogen-bond geometry (Å, °)

*D*—H⋯*A*	*D*—H	H⋯*A*	*D*⋯*A*	*D*—H⋯*A*
C11—H11*A*⋯O2	0.93	2.27	2.9153 (12)	126
C2—H2*A*⋯O5^i^	0.93	2.48	3.2820 (13)	145
C14—H14*A*⋯O4^ii^	0.93	2.46	3.3846 (12)	175
C21—H21*A*⋯O2^iii^	0.96	2.55	3.5064 (14)	173

Symmetry codes: (i) 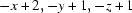; (ii) 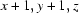; (iii) 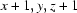.

**Fig. 1.**
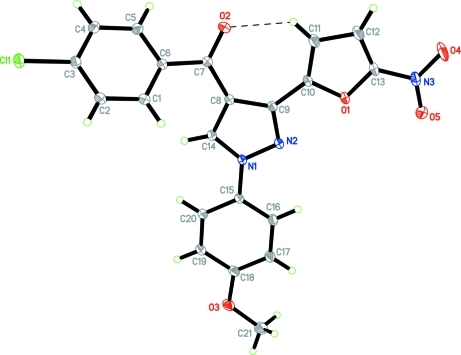
The molecular structure of (I), showing 50% probability displacement ellipsoids for non-H atoms. An intramolecular hydrogen bond is shown as dashed line.

**Fig. 2.**
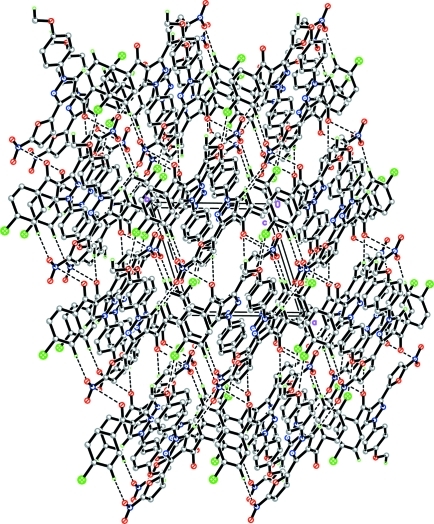
The crystal structure of (I), viewed along the c axis, showing the three-dimensional extended network. Hydrogen atoms not involved in intermolecular interactions (dashed lines) have been omitted for clarity.

## supplementary crystallographic information

### Comment

The pyrazole nucleus constitutes an interesting class of organic compound with diverse chemical applications. They possess anti-pyretic, anti-tumor, tranquilizing and herbicidal activities. Sydnones are easily accessible aromatic compounds and versatile synthetic intermediates with a masked azomethine imine unit. The 1,3-dipolar cycloaddition reaction with various dipolarophiles offers a convenient synthetic route for the preparation of pyrazole derivatives and has been studied extensively (Rai & Kalluraya, 2006; Rai *et al.*, 2008). The incorporation of 5-nitrofuran moiety into various heterocyclic systems has found to increase their biological activities. We have reported a few heterocyclic systems carrying 5-nitrofuran moiety as potent anti-microbial agents (Hedge *et al.*, 2006). In continuation of our studies on 1,3-dipolar cycloaddition reactions of sydnones with dipolarphiles carrying nitrofuran moiety (Kalluraya *et al.*, 1994), we herein report the crystal structure of the above pyrazole compound.

In the title pyrazole compound, an intramolecular C11—H11A···O2 hydrogen bond (Table 1) generates a seven-membered ring, producing an *S*(7) ring motif (Fig. 1, Bernstein *et al.*, 1995). The furan (C10-C13/O1) and pyrazole (C8/C9/N2/N1/C14) rings are essentially planar, with maximum deviations of 0.003 (1) and 0.004 (1) Å, respectively, at atoms O1 and N2. These two rings are coplanar to one another, making a dihedral angle of 3.06 (10)° between them. The pyrazole ring is inclined at dihedral angles of 47.59 (4) and 7.27 (4)°, respectively, with the mean planes through 4-chlorophenyl (C1-C6/Cl1) and 4-methoxyphenyl (C15-C20/O3/C21) groups. The nitro group is coplanar with the attached furan ring, as indicated by the dihedral angle formed of 2.03 (12)°. The bond lengths and angles are comparable to those observed in closely related pyrazole structures (Goh *et al.*, 2009*a,b*, 2010).

In the crystal structure, intermolecular C2—H2A···O5, C14—H14A···O4 and C21—H21A···O2 hydrogen bonds (Table 1) link neighbouring molecules into a three-dimensional extended network. The interesting feature of the crystal structure is the short intermolecular Cl1···O3 [3.0844 (9) Å, symmetry code: -x+3, -y+2, -z+1] and O2···N3 [2.8546 (12) Å, symmetry code: -x+1, -y+1, -z+1] interactions which are shorter than the sum of the van der Waals radii of the relevant atoms. The crystal structure is further stabilized by the weak intermolecular π–π interactions involving the pyrazole ring [*Cg*1···*Cg*1 = 3.4367 (6) Å; symmetry code: -x+2, -y+1, -z+1].

### Experimental

3-(p-Anisyl)sydnone (0.01 mol) and 1-(p-chlorophenyl)-3-(5-nitro-2-furyl)-2-propyn-1-one (0.01 mol) were dissolved in dry xylene (10 ml) and refluxed for 4 h. After completion of the reaction, the solvent was removed by distillation under reduced pressure. The crude product obtained was purified by recrystallization from a mixture of ethanol and DMF. The solid obtained was collected by filtration, washed with ethanol and dried.  Orange blocks of (I) were obtained from a 1:2 mixture of ethanol and DMF by slow evaporation.

### Refinement

All the hydrogen atoms were placed in their calculated positions, with C—H = 0.93 or 0.96 Å, and refined using a riding model with *U*_iso_ = 1.2 or 1.5 *U*_eq_(C). A rotating group model was used for the methyl group.

### Crystal data

**Table:**

C_21_H_14_ClN_3_O_5_	*Z* = 2
*M**_r_* = 423.80	*F*(000) = 436
Triclinic, *P*1	*D*_x_ = 1.513 Mg m^−^^3^
Hall symbol: -P 1	Mo *K*α radiation, λ = 0.71073 Å
*a* = 9.5589 (8) Å	Cell parameters from 9944 reflections
*b* = 9.6603 (8) Å	θ = 2.6–37.6°
*c* = 10.6401 (9) Å	µ = 0.25 mm^−^^1^
α = 95.523 (2)°	*T* = 100 K
β = 91.074 (2)°	Block, orange
γ = 107.706 (2)°	0.35 × 0.30 × 0.15 mm
*V* = 930.44 (13) Å^3^	

### Data collection

**Table:**

Bruker SMART APEX DUO CCD diffractometer	8076 independent reflections
Radiation source: fine-focus sealed tube	7107 reflections with *I* > 2σ(*I*)
graphite	*R*_int_ = 0.026
φ and ω scans	θ_max_ = 35.0°, θ_min_ = 2.2°
Absorption correction: multi-scan (*SADABS*; Bruker, 2009)	*h* = −15→15
*T*_min_ = 0.919, *T*_max_ = 0.963	*k* = −13→15
31674 measured reflections	*l* = −17→17

### Refinement

**Table:**

Refinement on *F*^2^	Primary atom site location: structure-invariant direct methods
Least-squares matrix: full	Secondary atom site location: difference Fourier map
*R*[*F*^2^ > 2σ(*F*^2^)] = 0.036	Hydrogen site location: inferred from neighbouring sites
*wR*(*F*^2^) = 0.146	H-atom parameters constrained
*S* = 1.13	*w* = 1/[σ^2^(*F*_o_^2^) + (0.095*P*)^2^ + 0.1254*P*] where *P* = (*F*_o_^2^ + 2*F*_c_^2^)/3
8076 reflections	(Δ/σ)_max_ = 0.001
272 parameters	Δρ_max_ = 0.87 e Å^−^^3^
0 restraints	Δρ_min_ = −0.70 e Å^−^^3^

### Special details

**Table:**

Experimental. The crystal was placed in the cold stream of an Oxford Cryosystems Cobra open-flow nitrogen cryostat (Cosier & Glazer, 1986) operating at 100.0 (1)K.
Geometry. All esds (except the esd in the dihedral angle between two l.s. planes) are estimated using the full covariance matrix. The cell esds are taken into account individually in the estimation of esds in distances, angles and torsion angles; correlations between esds in cell parameters are only used when they are defined by crystal symmetry. An approximate (isotropic) treatment of cell esds is used for estimating esds involving l.s. planes.
Refinement. Refinement of F^2^ against ALL reflections. The weighted R-factor wR and goodness of fit S are based on F^2^, conventional R-factors R are based on F, with F set to zero for negative F^2^. The threshold expression of F^2^ > 2sigma(F^2^) is used only for calculating R-factors(gt) etc. and is not relevant to the choice of reflections for refinement. R-factors based on F^2^ are statistically about twice as large as those based on F, and R- factors based on ALL data will be even larger.

### Fractional atomic coordinates and isotropic or equivalent isotropic displacement parameters (Å^2^)

**Table:**

	*x*	*y*	*z*	*U*_iso_*/*U*_eq_	
Cl1	1.29464 (2)	1.14454 (3)	0.06814 (2)	0.02139 (7)	
O1	0.61940 (7)	0.32154 (7)	0.56102 (7)	0.01521 (12)	
O2	0.71984 (7)	0.69219 (8)	0.30892 (7)	0.01788 (13)	
O3	1.40488 (8)	0.71448 (9)	1.04135 (7)	0.02060 (14)	
O4	0.30019 (9)	0.02849 (9)	0.52238 (8)	0.02777 (17)	
O5	0.47299 (9)	0.09317 (9)	0.67232 (9)	0.02794 (18)	
N1	0.99713 (8)	0.64428 (8)	0.64105 (7)	0.01227 (12)	
N2	0.87287 (8)	0.52812 (8)	0.62820 (7)	0.01314 (12)	
N3	0.41817 (9)	0.10960 (9)	0.57182 (8)	0.01796 (14)	
C1	1.11008 (9)	0.82550 (10)	0.28130 (8)	0.01484 (14)	
H1A	1.1386	0.7582	0.3241	0.018*	
C2	1.21148 (9)	0.92103 (10)	0.21216 (9)	0.01643 (15)	
H2A	1.3069	0.9161	0.2064	0.020*	
C3	1.16768 (9)	1.02368 (10)	0.15196 (8)	0.01521 (14)	
C4	1.02482 (10)	1.03149 (10)	0.15658 (9)	0.01617 (15)	
H4A	0.9977	1.1017	0.1167	0.019*	
C5	0.92349 (9)	0.93211 (10)	0.22196 (8)	0.01508 (14)	
H5A	0.8266	0.9332	0.2228	0.018*	
C6	0.96547 (9)	0.83057 (9)	0.28646 (8)	0.01292 (13)	
C7	0.84764 (9)	0.72576 (9)	0.35099 (8)	0.01295 (13)	
C8	0.88513 (9)	0.66693 (9)	0.46450 (8)	0.01251 (13)	
C9	0.80310 (8)	0.54115 (9)	0.52175 (8)	0.01230 (13)	
C10	0.66580 (9)	0.42891 (9)	0.48123 (8)	0.01289 (13)	
C11	0.56829 (10)	0.40179 (10)	0.37915 (8)	0.01690 (15)	
H11A	0.5753	0.4580	0.3119	0.020*	
C12	0.45459 (10)	0.27164 (11)	0.39557 (9)	0.01852 (16)	
H12A	0.3722	0.2250	0.3421	0.022*	
C13	0.49222 (9)	0.23009 (9)	0.50622 (9)	0.01568 (15)	
C14	1.00849 (9)	0.72922 (9)	0.54574 (8)	0.01321 (14)	
H14A	1.0848	0.8137	0.5363	0.016*	
C15	1.09929 (9)	0.66115 (9)	0.74528 (8)	0.01229 (13)	
C16	1.07607 (9)	0.55323 (10)	0.82671 (8)	0.01586 (15)	
H16A	0.9930	0.4716	0.8143	0.019*	
C17	1.17703 (10)	0.56696 (11)	0.92700 (9)	0.01755 (15)	
H17A	1.1624	0.4936	0.9805	0.021*	
C18	1.29982 (9)	0.69077 (10)	0.94696 (8)	0.01540 (14)	
C19	1.32113 (9)	0.80016 (10)	0.86573 (9)	0.01607 (15)	
H19A	1.4019	0.8839	0.8800	0.019*	
C20	1.22302 (9)	0.78476 (9)	0.76433 (8)	0.01446 (14)	
H20A	1.2393	0.8564	0.7091	0.017*	
C21	1.39244 (12)	0.60104 (13)	1.12158 (10)	0.0247 (2)	
H21A	1.4770	0.6276	1.1793	0.037*	
H21B	1.3867	0.5115	1.0709	0.037*	
H21C	1.3053	0.5879	1.1684	0.037*	

### Atomic displacement parameters (Å^2^)

**Table:**

	*U*^11^	*U*^22^	*U*^33^	*U*^12^	*U*^13^	*U*^23^
Cl1	0.01544 (10)	0.02511 (12)	0.01991 (12)	−0.00141 (8)	0.00097 (7)	0.00950 (8)
O1	0.0108 (2)	0.0127 (3)	0.0197 (3)	−0.0006 (2)	0.0000 (2)	0.0044 (2)
O2	0.0113 (2)	0.0195 (3)	0.0219 (3)	0.0026 (2)	−0.0025 (2)	0.0051 (2)
O3	0.0142 (3)	0.0274 (4)	0.0167 (3)	0.0004 (3)	−0.0036 (2)	0.0065 (3)
O4	0.0233 (3)	0.0221 (3)	0.0248 (4)	−0.0114 (3)	0.0003 (3)	−0.0004 (3)
O5	0.0186 (3)	0.0246 (4)	0.0393 (5)	0.0009 (3)	−0.0044 (3)	0.0172 (3)
N1	0.0098 (3)	0.0114 (3)	0.0141 (3)	0.0005 (2)	−0.0005 (2)	0.0027 (2)
N2	0.0099 (3)	0.0119 (3)	0.0157 (3)	0.0003 (2)	−0.0003 (2)	0.0025 (2)
N3	0.0147 (3)	0.0128 (3)	0.0234 (4)	−0.0004 (2)	0.0030 (3)	0.0023 (3)
C1	0.0117 (3)	0.0165 (3)	0.0162 (3)	0.0037 (3)	−0.0007 (2)	0.0037 (3)
C2	0.0111 (3)	0.0196 (4)	0.0177 (4)	0.0027 (3)	−0.0002 (3)	0.0045 (3)
C3	0.0131 (3)	0.0165 (3)	0.0140 (3)	0.0010 (3)	0.0003 (2)	0.0034 (3)
C4	0.0156 (3)	0.0177 (4)	0.0161 (3)	0.0053 (3)	0.0015 (3)	0.0055 (3)
C5	0.0134 (3)	0.0173 (3)	0.0159 (3)	0.0057 (3)	0.0018 (2)	0.0049 (3)
C6	0.0114 (3)	0.0133 (3)	0.0137 (3)	0.0027 (2)	0.0002 (2)	0.0028 (2)
C7	0.0109 (3)	0.0130 (3)	0.0146 (3)	0.0028 (2)	−0.0001 (2)	0.0026 (2)
C8	0.0102 (3)	0.0123 (3)	0.0140 (3)	0.0017 (2)	−0.0001 (2)	0.0025 (2)
C9	0.0098 (3)	0.0115 (3)	0.0150 (3)	0.0021 (2)	0.0008 (2)	0.0020 (2)
C10	0.0100 (3)	0.0121 (3)	0.0150 (3)	0.0010 (2)	0.0014 (2)	0.0016 (2)
C11	0.0153 (3)	0.0168 (4)	0.0143 (3)	−0.0012 (3)	−0.0006 (3)	0.0011 (3)
C12	0.0159 (3)	0.0183 (4)	0.0154 (4)	−0.0027 (3)	−0.0003 (3)	−0.0009 (3)
C13	0.0123 (3)	0.0127 (3)	0.0185 (4)	−0.0012 (3)	0.0018 (3)	0.0008 (3)
C14	0.0112 (3)	0.0125 (3)	0.0146 (3)	0.0011 (2)	−0.0002 (2)	0.0030 (2)
C15	0.0101 (3)	0.0124 (3)	0.0134 (3)	0.0019 (2)	0.0001 (2)	0.0017 (2)
C16	0.0140 (3)	0.0154 (3)	0.0151 (3)	−0.0008 (3)	−0.0009 (3)	0.0042 (3)
C17	0.0152 (3)	0.0198 (4)	0.0153 (4)	0.0008 (3)	−0.0007 (3)	0.0061 (3)
C18	0.0117 (3)	0.0197 (4)	0.0133 (3)	0.0027 (3)	−0.0002 (2)	0.0021 (3)
C19	0.0116 (3)	0.0159 (3)	0.0186 (4)	0.0011 (3)	−0.0013 (3)	0.0021 (3)
C20	0.0112 (3)	0.0130 (3)	0.0180 (4)	0.0016 (2)	−0.0011 (2)	0.0031 (3)
C21	0.0208 (4)	0.0327 (5)	0.0192 (4)	0.0042 (4)	−0.0033 (3)	0.0096 (4)

### Geometric parameters (Å, °)

**Table:**

Cl1—C3	1.7351 (9)	C7—C8	1.4662 (12)
O1—C13	1.3488 (11)	C8—C14	1.3892 (11)
O1—C10	1.3774 (10)	C8—C9	1.4294 (11)
O2—C7	1.2282 (10)	C9—C10	1.4516 (11)
O3—C18	1.3593 (11)	C10—C11	1.3696 (12)
O3—C21	1.4313 (13)	C11—C12	1.4180 (13)
O4—N3	1.2343 (11)	C11—H11A	0.9300
O5—N3	1.2270 (12)	C12—C13	1.3563 (13)
N1—C14	1.3503 (11)	C12—H12A	0.9300
N1—N2	1.3574 (10)	C14—H14A	0.9300
N1—C15	1.4275 (11)	C15—C16	1.3883 (12)
N2—C9	1.3393 (11)	C15—C20	1.3969 (12)
N3—C13	1.4200 (12)	C16—C17	1.3942 (12)
C1—C2	1.3949 (12)	C16—H16A	0.9300
C1—C6	1.3998 (12)	C17—C18	1.3933 (13)
C1—H1A	0.9300	C17—H17A	0.9300
C2—C3	1.3906 (12)	C18—C19	1.3987 (13)
C2—H2A	0.9300	C19—C20	1.3828 (12)
C3—C4	1.3919 (12)	C19—H19A	0.9300
C4—C5	1.3899 (13)	C20—H20A	0.9300
C4—H4A	0.9300	C21—H21A	0.9600
C5—C6	1.3975 (12)	C21—H21B	0.9600
C5—H5A	0.9300	C21—H21C	0.9600
C6—C7	1.4948 (12)		
			
C13—O1—C10	105.18 (7)	O1—C10—C9	114.95 (7)
C18—O3—C21	117.66 (8)	C10—C11—C12	106.87 (8)
C14—N1—N2	112.41 (7)	C10—C11—H11A	126.6
C14—N1—C15	128.09 (7)	C12—C11—H11A	126.6
N2—N1—C15	119.46 (7)	C13—C12—C11	104.95 (8)
C9—N2—N1	105.18 (7)	C13—C12—H12A	127.5
O5—N3—O4	124.52 (9)	C11—C12—H12A	127.5
O5—N3—C13	119.17 (8)	O1—C13—C12	112.92 (8)
O4—N3—C13	116.31 (9)	O1—C13—N3	116.80 (8)
C2—C1—C6	120.23 (8)	C12—C13—N3	130.27 (8)
C2—C1—H1A	119.9	N1—C14—C8	107.30 (7)
C6—C1—H1A	119.9	N1—C14—H14A	126.3
C3—C2—C1	118.94 (8)	C8—C14—H14A	126.3
C3—C2—H2A	120.5	C16—C15—C20	120.14 (8)
C1—C2—H2A	120.5	C16—C15—N1	119.68 (7)
C2—C3—C4	121.82 (8)	C20—C15—N1	120.17 (7)
C2—C3—Cl1	118.96 (7)	C15—C16—C17	120.14 (8)
C4—C3—Cl1	119.22 (7)	C15—C16—H16A	119.9
C5—C4—C3	118.60 (8)	C17—C16—H16A	119.9
C5—C4—H4A	120.7	C18—C17—C16	119.84 (8)
C3—C4—H4A	120.7	C18—C17—H17A	120.1
C4—C5—C6	120.81 (8)	C16—C17—H17A	120.1
C4—C5—H5A	119.6	O3—C18—C17	124.49 (8)
C6—C5—H5A	119.6	O3—C18—C19	115.85 (8)
C5—C6—C1	119.53 (8)	C17—C18—C19	119.66 (8)
C5—C6—C7	116.77 (7)	C20—C19—C18	120.48 (8)
C1—C6—C7	123.58 (7)	C20—C19—H19A	119.8
O2—C7—C8	120.95 (8)	C18—C19—H19A	119.8
O2—C7—C6	119.13 (8)	C19—C20—C15	119.70 (8)
C8—C7—C6	119.91 (7)	C19—C20—H20A	120.1
C14—C8—C9	104.13 (7)	C15—C20—H20A	120.1
C14—C8—C7	126.36 (8)	O3—C21—H21A	109.5
C9—C8—C7	129.40 (7)	O3—C21—H21B	109.5
N2—C9—C8	110.98 (7)	H21A—C21—H21B	109.5
N2—C9—C10	117.95 (7)	O3—C21—H21C	109.5
C8—C9—C10	131.03 (8)	H21A—C21—H21C	109.5
C11—C10—O1	110.07 (7)	H21B—C21—H21C	109.5
C11—C10—C9	134.98 (8)		
			
C14—N1—N2—C9	−0.53 (9)	O1—C10—C11—C12	0.31 (10)
C15—N1—N2—C9	−178.56 (7)	C9—C10—C11—C12	179.62 (9)
C6—C1—C2—C3	1.91 (13)	C10—C11—C12—C13	−0.05 (10)
C1—C2—C3—C4	−1.44 (14)	C10—O1—C13—C12	0.43 (10)
C1—C2—C3—Cl1	178.93 (7)	C10—O1—C13—N3	−178.49 (7)
C2—C3—C4—C5	−0.89 (14)	C11—C12—C13—O1	−0.24 (11)
Cl1—C3—C4—C5	178.74 (7)	C11—C12—C13—N3	178.50 (9)
C3—C4—C5—C6	2.78 (14)	O5—N3—C13—O1	−1.02 (13)
C4—C5—C6—C1	−2.31 (13)	O4—N3—C13—O1	178.36 (8)
C4—C5—C6—C7	−178.41 (8)	O5—N3—C13—C12	−179.72 (10)
C2—C1—C6—C5	−0.08 (13)	O4—N3—C13—C12	−0.34 (15)
C2—C1—C6—C7	175.74 (8)	N2—N1—C14—C8	0.08 (9)
C5—C6—C7—O2	27.96 (12)	C15—N1—C14—C8	177.90 (7)
C1—C6—C7—O2	−147.96 (9)	C9—C8—C14—N1	0.37 (9)
C5—C6—C7—C8	−150.78 (8)	C7—C8—C14—N1	177.02 (8)
C1—C6—C7—C8	33.30 (12)	C14—N1—C15—C16	−172.85 (8)
O2—C7—C8—C14	−156.41 (9)	N2—N1—C15—C16	4.84 (12)
C6—C7—C8—C14	22.30 (12)	C14—N1—C15—C20	6.33 (13)
O2—C7—C8—C9	19.38 (14)	N2—N1—C15—C20	−175.98 (7)
C6—C7—C8—C9	−161.90 (8)	C20—C15—C16—C17	−0.74 (13)
N1—N2—C9—C8	0.76 (9)	N1—C15—C16—C17	178.45 (8)
N1—N2—C9—C10	178.64 (7)	C15—C16—C17—C18	1.34 (14)
C14—C8—C9—N2	−0.72 (9)	C21—O3—C18—C17	3.45 (14)
C7—C8—C9—N2	−177.23 (8)	C21—O3—C18—C19	−176.00 (9)
C14—C8—C9—C10	−178.24 (8)	C16—C17—C18—O3	−179.70 (8)
C7—C8—C9—C10	5.26 (15)	C16—C17—C18—C19	−0.27 (14)
C13—O1—C10—C11	−0.45 (9)	O3—C18—C19—C20	178.05 (8)
C13—O1—C10—C9	−179.91 (7)	C17—C18—C19—C20	−1.43 (13)
N2—C9—C10—C11	−177.28 (9)	C18—C19—C20—C15	2.03 (13)
C8—C9—C10—C11	0.10 (16)	C16—C15—C20—C19	−0.95 (13)
N2—C9—C10—O1	2.01 (11)	N1—C15—C20—C19	179.87 (8)
C8—C9—C10—O1	179.38 (8)		

### Hydrogen-bond geometry (Å, °)

**Table:**

*D*—H···*A*	*D*—H	H···*A*	*D*···*A*	*D*—H···*A*
C11—H11A···O2	0.93	2.27	2.9153 (12)	126
C2—H2A···O5^i^	0.93	2.48	3.2820 (13)	145
C14—H14A···O4^ii^	0.93	2.46	3.3846 (12)	175
C21—H21A···O2^iii^	0.96	2.55	3.5064 (14)	173

Symmetry codes: (i) −*x*+2, −*y*+1, −*z*+1; (ii) *x*+1, *y*+1, *z*; (iii) *x*+1, *y*, *z*+1.
